# Autism Mental Status Examination (AMSE): A Valid Instrument in the Evaluation of Pre-school Children with Suspected Autism Spectrum Disorders?

**DOI:** 10.1007/s10803-019-04012-1

**Published:** 2019-05-04

**Authors:** Mats Cederlund

**Affiliations:** 1 Present Address: Child and Adolescent Habilitation Unit, Regnbågsgatan 1A, 41755 Gothenburg, Sweden; 2 Child- and Adolescent Clinic, NU-Hospital Group, NÄL, 461 85 Trollhättan, Sweden

**Keywords:** Autism mental status examination, DISCO-11, Autism spectrum disorders, Vineland adaptive behavior skills II parent/caregiver rating questionnaire

## Abstract

In this study the Autism Mental Status Exam (AMSE) was validated towards ICD-10 Autism Spectrum Diagnoses (ASD) based on an interview with the Diagnostic Interview for Social and Communication Disorders (DISCO-11) with parent(s)/caregiver(s) in a group of 124 children referred for assessment to a clinical assessment unit for pre-school children. The results from the study indicates a *Fair* relation across the AMSE score and ICD-10 Autism Spectrum Disorder (ASD). AMSE mean score for children not fulfilling criteria for an ASD at the assessment was significantly lower compared to the AMSE mean score for children who acquired an ASD diagnosis in the study. In addition, Vineland-II Parent/caregiver rating questionnaire GAF standard scores showed a reversed correlation to the AMSE mean scores (i.e. higher AMSE mean scores were related to lower Vineland-II GAF standard scores and vice versa).

## Introduction

The screening and assessment of children with a suspected Autism Spectrum Disorder (ASD) has been a matter of research for several decades, and a number of screening and supplementary instruments have been produced to facilitate the assessment. The CHecklist for Autism in Toddlers (CHAT) (Baron-Cohen et al. 1992), further developed by Robins et al. (2001) to the Modified-CHecklist for Autism in Toddlers (M-CHAT) for level one screening (i.e. screening as part of a routine developmental surveillance) for use in children 16–48 months of age with the aim to identify children in need of further assessment. In a study by Øien et al. (2018a) children having passed the M-CHAT 6-critical-item criterion at 18 months, albeit later being diagnosed with ASD (false-negative M-CHAT), were found to have exhibited distinct delays in social, communication, and motor skills reported from caregiver(s), with differences being more pronounced in girls, compared to children who had a true-negative M-CHAT at 18 months of age. Possible reasons for a false-negative M-CHAT include: (1) difficulties for the caregiver(s) to map the specific behavioral markers considered in their child’s real-life behaviors; (2) difficulties for the caregiver(s) in understanding some of the phenomenology of more specific or rare behaviors related to ASD; (3) the fact that M-CHAT does not provide opportunities for graded response; (4) symptoms related to ASD might be expressed differently in early childhood depending on the child’s specific verbal, and non-verbal skills, or temperamental characteristics. However, it must be emphasized here that the M-CHAT has been further revised to the M-CHAT R/F, where the authors recommend a follow-up interview to provide greater utility (Robins et al. 2014). The next level of questionnaires, the level two screening questionnaires (e.g. the Autism Behavior Checklist (ABC) (Krug et al. 1980), the Autism Spectrum Screening Questionnaire (ASSQ) (Ehlers et al. 1999), and the Social Communication Questionnaire (SCQ) (Rutter et al. 2003), focuses on the evaluation of autistic signs and symptoms present in an individual suspected of having an ASD. The ABC questionnaire is easy to administer, and covers all main problematic areas included in ASD, albeit was originally developed for the assessment of autistic symptoms in individuals with a pronounced intellectual disability, and is hence less assigned for children with near normal or normal intelligence. In contrast, the ASSQ was developed to screen for autistic symptoms in school children with normal intelligence, and has been found to be a good screening instrument for that group, with a sensitivity rate of 91%, and a specificity rate of 83%, albeit is less useful in children below 5 years of age (Posserud et al. 2009). The SCQ, which is based on the ADI-R, was developed for use from 4 years of age, and aims at collecting information about the child’s possible difficulties within the autism spectrum as a base for further assessment (Rutter et al. 2003).

The Autism Diagnostic Observational Schedule (ADOS/ADOS-2) was developed with the purpose to structure the observation of an individual with suspected ASD in a clinical setting (Lord et al. 2001; Lord et al. 2012a, b). The ADOS is today a main instrument in assessment schemes for ASD all over the world. However the ADOS requires specific professional training prior to the use of the instrument, and it needs a adequate clinical setting in which to be performed.

Semi-quantitative interviews (e.g. Autism Diagnostic Interview (ADI/ADI-R) (LeCouteur et al. 1989; Lord et al. 1994), and Diagnostic Interview for Social and Communication Disorders (DISCO) (Wing et al. 2002)) are instruments that combines the information retrieved from the parent(s)/caregiver(s) with the investigating professional’s view of the child, in order to provide a complete assessment of the child as possible. The ADI-R, and the DISCO-11 share a common origin, as they were both originally developed from the Handicaps Behavior and Skills schedule (HBS) (Wing and Gould 1978). In contrast to the ADI, the DISCO has a neurodevelopmental perspective and includes items concerning psychiatric problems. The DISCO-11 has been validated towards the ADOS with sufficient agreeability **(**sensitivity 93%, and specificity 79%) (Maljars et al. 2012). In addition, the DISCO-10 was validated towards the ADI-R, and the authors found an excellent overall agreement across the instruments according to the Landis and Koch criteria (Landis and Koch 1977; Nygren et al. 2009). However, both these semi-quantitative interviews require special training prior to use of the respective instrument, and are time-consuming to perform.

With the purpose to establish an easy accessible and valid tool in the investigation of individuals referred for neuropsychiatric assessment, including both observational items and information retrieved from parent(s)/caregiver(s), Grodberg et al. (2012) developed the Autism Mental Status Examination (AMSE). The AMSE was developed to structure the evaluation of autistic signs and symptoms, and hence improving the judgment for clinicians assessing individuals with a suspected ASD. The AMSE has been validated towards the ADI-R, as well as the ADOS/ADOS 2, and was proven to have a good reliability (i.e. a sensitivity rate as well as a specificity rate exceeding 80%), towards these instruments according to the validation norms of Cicchetti et al. (1995). In addition, when the AMSE was validated towards DSM-5 ASD criteria (American Psychiatric Association 2013) the authors found a sensitivity rate of 93% and a specificity rate of 100% for an AMSE cut off score of 6p in the children included in their study (Grodberg et al. 2012, 2014; Grodberg et al. 2016). The AMSE has previously only been used in Sweden to a limited extent. However since it captures the core symptoms of ASD, is supported by highly valid research data, and is easy to administer the author, who has used it with good experience in clinical work, decided to perform a study of it in pre-school children referred for neuropsychiatric assessment.

In the current study the primary aim was to validate the AMSE towards the International statistical Classification of Diseases—Tenth Edition (ICD-10) ASD diagnoses (i.e. Autism, Atypical Autism, and Asperger Syndrome) (World Health Organisation 1992) in children referred for neuropsychiatric assessment to local team for pre-school children at the NU-Hospital group in Sweden. To acquire the essential information required for the ICD-10 ASD diagnoses the DISCO-11 was selected, since it, in contrast to the ADI-R, had not been validated towards the AMSE prior to this study. The secondary aim for this study was to assess the participating children on the Vineland Adaptive Behavior Scales-II Parent/caregiver rating questionnaire (Sparrow et al. 2005) to achieve the child’s General Adaptive Functioning (GAF), and adaptive functioning in the subdomains covered by the Vineland-II Parent/caregiver rating questionnaire (i.e. Communication, Daily Living Skills (DLS), Socialization, and Motor skills). In addition, the participants intellectual/developmental ability, and expressive language ability were analyzed in comparison to the AMSE and the Vineland-II Parent/caregiver rating questionnaire.

## Methods

### Participants

**Fig. 1:**
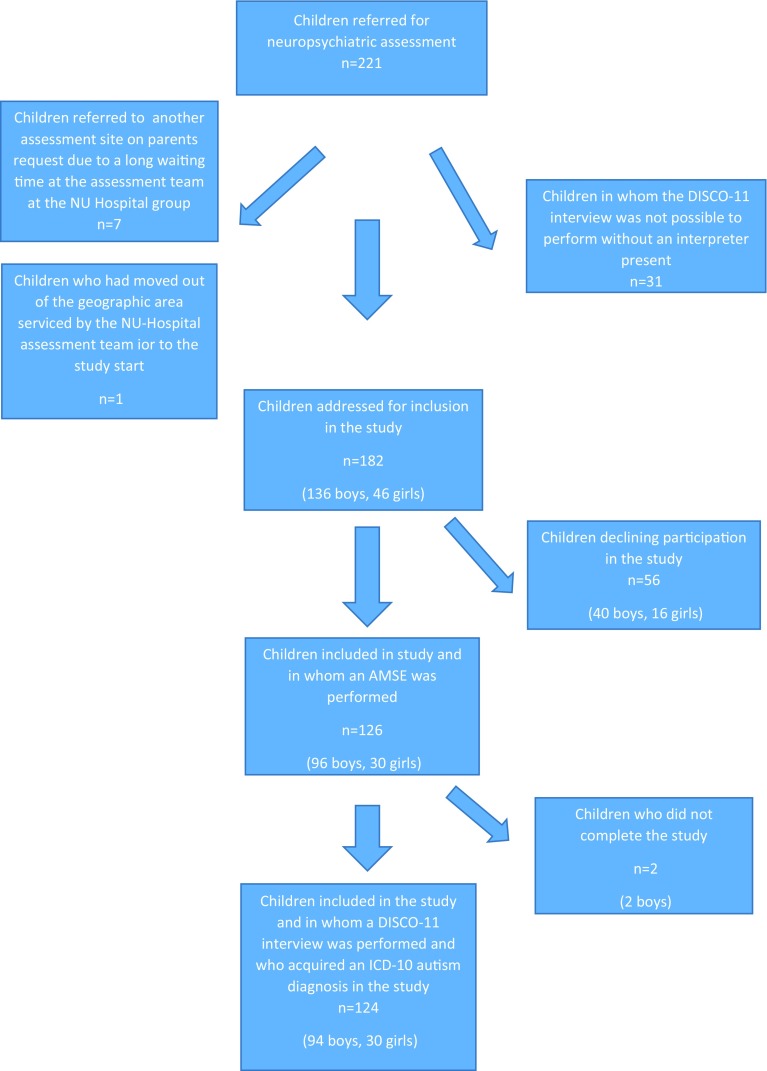
Flow chart for children referred for neuropsychiatric assessment to the team for pre-school children at the NU Hospital group in Trollhattan, Sweden, and hence being potential participants in the present study

All children referred consecutively to the local assessment team for pre-school children at the NU Hospital Group in Trollhättan, Sweden for neuropsychiatric assessment from April 2014 to June 2016 were potential participants in the AMSE study. Se Flow chart in Fig. 1 for further information. As visualized in the flow chart in Fig. 1, 182 children remained for inclusion in the study after the excluded children were removed.

All the parent(s)/caregiver(s) of these 182 children were addressed by a letter with information about the present study and a form to be signed, and returned by mail in a prepaid envelope, if they decided for their child to participate in the study. If custody over the child was shared both parents *had* to sign the consent. At the first visit to the clinic the parent(s), who had not sent their consent by mail or delivered it immediately at the visit, were asked if they had received the invitation letter, and if they specifically asked for additional information about the study before making their decision, this was given by the investigator. Informed consent was obtained for all individual participants included in the study, where the assessments were performed from January 2015 to March 2017.

One child was included in the study although the DISCO-11 interview with the parents had to be performed with an interpreter present, since the parents did not master Swedish good enough to perform a DISCO-11 interview without an interpreter present.

Of the 182 children addressed for inclusion in the study 126 children (69%) agreed to participate, and 56 children declined participation. The group of children who were included in the study had a mean age of 49.4 (SD15.6) months at referral, which was not significantly different compared to a mean age of 50.8 (SD 16.5) months at referral for the children where the parent(s)/caregiver(s) declined participation. According to the agreement with the Ethics committee at Gothenburg University the reason for declining to participate was not further discussed with the parents who declined participation.

There were 96 boys and 30 girls included in the study, giving a 3.2:1 male:female ratio, which was not significantly different from the group of children declining participation were there were 40 boys and 16 girls, giving a 2.5:1 male:female ratio. There were 2 pairs of siblings in the investigated group (sister, brother; 2 brothers), and one child was adopted from Lithuania. Further data about the participating children are presented in Table 1.

**Table 1:** Demographic data concerning the study participants

	All (n = 126)	ICD-10 Autism (n = 89)	ICD-10 Atypical autism (n = 14)	ICD-10 AS (n = 3)	No ICD-10 ASD diagnosis (n = 18)
Girls (%)	30 (23.8%)	19 (15.1%)	5 (4.0%)	1 (0.8%)	5 (4.0%)
Boys (%)*/**	96 (76.2%)	70 (55.6%)	9 (7.1%)	2 (1.6%)	13 (10.3%)
Mean age all participants in in months (SD)	54.4 (15.8)	53.8 (16.1)	48.3 (16.8)	72.7 (11.1)	57.2 (14.6)
Median (min; max)	55 (11; 94)	56 (11; 94)	47 (30; 81)	74 (61; 83)	56 (24; 85)
Mean age girls in months (SD)	50.9 (15.4)	51.6 (15.8)	43.8 (10.2)	74.0 (0)	51.0 (17.2)
Median (min; max)	54.5 (24; 94)	56 (28; 73)	44 (30; 54)	74 (74)	55 (24; 71)
Mean age boys in months (SD)	55.4 (15.8)	54.3 (16.2)	51.4 (14.3)	72.0 (15.6)	59.5 (13.4)
Median (min; max)	56 (11; 94)	55.5 (11; 94)	48 (36; 81)	72 (61; 83)	59 (35; 85)
Born in Sweden*/**	119 (94.4%)	83 (69.7%)	13 (10.9%)	3 (2.5%)	18 (15.1%)
Born abroad	7 (5.6%)	6 (85.7%)	1 (14.3%)	0 (0%)	0 (0%)
Prematurely born girls	4 (13.3%)	4 (100%)	0(0%)	0 (0%)	0 (0%)
Prematurely born boys*	8 (8.3%)	4 (50.0%)	2 (25.0%)	0 (0%)	1 (12.5%)

*One prematurely born boy was not diagnosed due to discontinuation of the study

**One boy born at term was not diagnosed due to discontinuation of the study

### Procedure

#### AMSE

The AMSE was performed by the investigator at the child’s visit to the clinic in all of the 126 participating individuals by the investigator. The maximum AMSE score was 16p for the children ≥ 37 months, and 14p for the children ≤ 36 months, since item 5 in the AMSE (Cannot take turns or topics and/or Unvaried or odd intonation) was considered difficult to score with reliable certainty in children ≤ 36 months of age, who in most cases had neither developed a speech available for the judging of unvaried or odd intonation nor being mentally old enough for taking turns or topics. The AMSE was scored by the investigator during or immediately after the child’s visit to the clinic, and the scoring sheet was filed directly after the scoring was performed and was not used again by the investigator until the processing of the study data was performed.

#### DISCO-11

The DISCO-11 was completed in 124 individuals (98%) at a separate visit to the clinic for the parent(s)/caregiver(s) approximately 1 month after the AMSE was scored. In two boys originally included in the study no DISCO-11 interview was performed. One of these boys was placed in foster care after the initial visit to the clinic, and the biological mother did not appear for the DISCO-11 interview, and could not be reached by telephone or letter to confirm discontinuation of the study. The other boy was in foster care when he was included in the study, albeit was later repatriated with the father, who originally signed the informed consent as the child’s single guardian, albeit after the repatriation the father did not want for the child to continue in the study.

At the DISCO-11 interview both parents were present in 81 cases (65%), only the mother in 33 cases (28%), and only the father in 9 cases (7%). In five cases parent(s) were accompanied by the child’s stepfather/stepmother or a grandparent.

All the received data from the DISCO-11 interview was transported into the DISCO-11 computer system in which the criteria for ICD-10 ASD diagnoses constitutes the algorithm, and the DISCO-11 computer system delivered the ICD-10 ASD diagnosis for which all required ICD-10 criteria was met.

#### Vineland-II Parent/Caregiver Rating Questionnaire

At the DISCO-11-interview the Vineland-II Parent/caregiver rating questionnaire was distributed to the parent(s)/caregiver(s) along with a prepaid envelope to be returned by mail to the investigator. In two cases the Vineland-II Parent/caregiver rating questionnaire was not distributed because these children were too young (approximately 1½ years of age) to be adequately assessed by the Vineland-II Parent/caregiver rating questionnaire, which is validated from 24 months of age. In addition the parents mentioned above who did not master Swedish well enough to be able to fill in the questionnaire by themselves did not receive the Vineland-II Parent/caregiver rating questionnaire. All in all 121 Vineland-II Parent/caregiver rating questionnaires were distributed, and 117 (97%) were returned after approximately two weeks in general. The scores from the Vineland-II Parent/caregiver rating questionnaire was processed in the Vineland-II data program by the investigator to achieve the standard scores for GAF, and the domains included in the Vineland-II Parent/caregiver rating questionnaire.

#### Intellectual Assessment and Classification

A psychological assessment had been performed in 112 individuals, however in 17 of these cases no intelligence/developmental test was performed. The vast majority (89 individuals) were tested with one of the Wechsler scales (i.e. Wechsler Preschool and Primary Scale of Intelligence—Third Edition [WPPSI-III (n = 25)], Wechsler Preschool and Primary Scale of Intelligence—Fourth Edition [WPPSI-IV (n = 55)], Wechsler Intelligence Scale for Children—Fourth Edition [WISC-IV (n = 5)], or Wechsler Nonverbal Scale of Ability [WNV (n = 4)]. For the remaining 6 individuals the Merrill-Palmer-R (n = 4) or Bayley Scales of Toddler Development (n = 2) were used. The participants where a test of intelligence/developmental level for some reason was not performed had their developmental/intellectual ability determined by their general functioning at the visit to the clinic and information concerning developmental abilities acquired from the DISCO-11 interview.

All the participating children were collapsed into groups according to the intellectual ability grading system presented in the DISCO-11: (1) Profound intellectual disability (IQ 0–19), (2) Severe intellectual disability (IQ 20–34), (3) Moderate intellectual disability (IQ 35–49), (4) Mild intellectual disability (IQ 50–69), (5) Intelligence below average (IQ 70–89), (6) Average intelligence (IQ 90–119, (7) Intelligence above average (IQ ≥ 120).

#### Language Ability Classification

The classification of the child´s language ability was based on the expressive language levels presented in the AMSE sheet (i.e. “Nonverbal”, “Single words”, “Phrases”, “Undeveloped sentences”, and “Can speak about another time and place”).

The investigating author who made the scoring of the AMSE had not received any specific training concerning the AMSE prior to the study, however the AMSE had been used by the author in the clinical setting for approximately 2 years prior to the study, and the author was hence familiar with the use of the AMSE before the study started.

All the DISCO-11 interviews were performed by the investigating author who has a long experience within the neuropsychiatric field and has used the DISCO-11 for more than 15 years in research as well as in clinical work.

Since the investigating author performed the AMSE, as well as the DISCO-11 interview this study was not conducted blind.

### Measures

#### Instruments Used

##### Autism Mental Status Examination—AMSE

The AMSE is a short standardized direct observational instrument that aims to improve the clinical judgment in the diagnostic process of ASD. It has been validated towards DSM-5 ASD diagnosis, as well as towards the ADOS/ADOS-2 and ADI-R (see Introduction). It structures the observation and documentation of eight items comprising social, communicative, and behavioral signs and symptoms of ASD that typically emerge throughout a neurodevelopmental evaluation. The AMSE structures direct observations by the investigator for all the eight items included, albeit in addition it provides opportunity to record clinical information received from the caregiver at the assessment. The first 3 items are scored solely by the investigator and relates to direct observations of the child, and item 4–8 are scored based both on the child´s presentation at the visit or by information from the caregiver. Each item is scored 0, 1 or 2 p depending on severity of symptoms as specified below. Observed symptoms or behavior for item 4–8 is scored higher (2p) compared to information provided only by the caregiver(s) (1p). The eight items include (1) Eye contact (observed) (≥ 3 s 0p, Fleeting 1p, None 2p), (2) Interest in others (observed) (Initiates interaction with Examiner 0p, Only passively responds 1p, No interest 2p), (3) Pointing skills (observed) (Can point/Gesture to object 0p, Only follows point 1p, None 2p), (4) Language (reported or observed) (Can speak about another time or place 0p, Single words/Phrases (≤ 3 words)/Undeveloped sentences 1p, Nonverbal 2p), (5) Pragmatics of language (reported or observed) (Not impaired (Not applicable) 0p, Cannot manage turns or topics/Unvaried or odd intonation, reported 1p, observed 2p) (6) Repetitive behaviors/stereotypy (reported or observed) None 0p, Compulsive behaviors/insists on routines 1p, Motor mannerisms/Echolalia/Stereotyped speech 2p), (7) Unusual or encompassing preoccupations (reported or observed) (none 0p, Present reported 1p, observed 2p), and (8) Unusual sensitivities (reported or observed) (None 0p, Heightened sensitivity/High pain threshold reported 1p, observed 2p) (Grodberg et al. 2012).

##### Diagnostic Interview for Social and Communication Disorders (DISCO-11)

A semi-structured instrument intended for interview with a person (parent/caregiver), who knows the individual well. The ICD-10 ASD criteria constitutes the algorithm of the DISCO-11 from which ICD-10 ASD diagnoses can be retrieved for *Ever* as well as *Current* situations.

The DISCO-11 has been proven to have excellent inter-rater and test–retest reliability according to the Landis and Koch (1977) criteria (Nygren et al. 2009). It includes a range of items intended to detect milder forms of ASD and has been validated for assigning diagnoses in the autism spectrum at a similar level to the ADI-R (See introduction). In addition, the DISCO-11 has a developmental perspective and is hence designed for use from early childhood (Wing et al. 2002; Wing et al. 2006). The agreement between DISCO-11 and ADOS has been found substantial with good criterion-related and convergent validity for children with normal intelligence or mild intellectual ability, however with a tendency for the DISCO-11 to be over inclusive in children with moderate to severe intellectual disability, giving an overall sensitivity rate of 93%, and a specificity rate of 79% (Maljars et al. 2012).

##### Vineland Adaptive Behavior Scales Second Edition (Vineland-II) Parent/Caregiver Rating Questionnaire

A parent/caregiver rating questionnaire that offers a comprehensive assessment of adaptive behaviour in three major areas: (1) Communication (receptive, expressive, written) (2) Daily Living Skills (DLS) (personal, domestic, community), and (3) Socialization (interpersonal relationships, play and leisure time, coping skills). In addition a Motor Skills domain is included. From the scores of these scales a General Adaptive Functioning (GAF) standard score can be derived (Sparrow et al. 2005).

#### Intelligence/Developmental Tests

##### Wechsler Preschool and Primary Scale of Intelligence–Third Edition (WPPSI-III)

An intelligence test designed for children 2 years 6 months–7 years 7 months, which provides subtests and composite scores that represents intellectual functioning in verbal and performance cognitive domains, as well as provide a composite score that represents a child’s general intellectual ability (Wechsler 2002).

##### Wechsler Preschool and Primary Scale of Intelligence—Fourth Edition (WPPSI-IV)

An intelligence test designed for children 2 years 6 months–7 years 7 months, which provides subtests and composite scores that represents intellectual functioning in verbal comprehension, visual spatial capacity, working memory, fluid reasoning, and processing speed, as well as provide a composite score that represents a child’s general intellectual ability (Wechsler 2012).

##### Wechsler Intelligence Scale for Children—Fourth Edition (WISC-IV)

An intelligence test designed for children 6 years–16 years 11 months, which provides subtests and composite scores that represents intellectual functioning in verbal comprehension, perceptual reasoning, working memory and processing speed, as well as provide a composite score that represents a child’s general intellectual ability (Wechsler 2003).

##### Wechsler Nonverbal Scale of Ability (WNV)

A Nonverbal measure of ability designed for culturally and linguistically diverse groups of children and young adults from 4 to 21 years 11 months, which provides subtests and composite scores that represents intellectual functioning in performance cognitive domains (Wechsler and Naglieri 2006).

##### Bayley Scales of Infant and Toddler Development—Third Edition

A developmental test for children 1–42 months covering Adaptive Behavior, Cognitive, Language, Motor, and Social-Emotional domains (Bayley 2006).

##### Merrill-Palmer—Revised Scales of Development

A developmental test for children 1 month–6:5 years consisting of a cognitive test battery containing three domains: Cognition, Fine Motor Skills and Receptive Language skills. In addition there are 3 additive scales including Memory, Processing Speed, and Visuo-motor ability (Roid and Sampers 2000).

## Statistical Methods

Descriptive statistics are given as mean, standard deviation, median, minimum and maximum for continuous variables and as numbers and percentages for categorical variables. For comparison between two groups Student two-sample *T* test was used for continuous variables, Chi square test for dichotomous variables and Mantel–Haenszel Chi square test for ordered categorical variables. Spearman correlation coefficient was used for all correlation analyses. Sensitivity, specificity, positive and negative predicted value were calculated for different AMSE cut offs. All significance tests were two-sided and conducted at the 5% significance level.

## Results

Of the 124 children where a DISCO-11 interview was performed, 106 children (81 boys, and 25 girls) had a *Current* ICD-10 ASD diagnosis giving a 3.2:1 male:female ratio. For the individuals having an intellectual ability within the normal intellectual distribution (i.e. IQ ≥ 70) the male:female ratio was 4.4:1 compared to 1.9:1 for the children with an intellectual ability ≤ IQ 70. Eighty-nine children (70 boys, 19 girls) acquired a *Current* ICD-10 Autism diagnosis. Fourteen children (9 boys, 5 girls) were found to have a *Current* ICD-10 Atypical autism diagnosis. Altogether, eighteen individuals fulfilled criteria for a *Current* ICD-10 diagnosis of AS, however in 15 of these cases criteria for a *Current* ICD-10 Autism (n = 12) or ICD-10 Atypical autism (n = 3) was also met, which took precedence over the ICD-10 AS diagnosis, leaving 3 children (2 boys, 1 girl) to be classified with a *Current* ICD-10 AS diagnosis. Eighteen individuals (13 boys, 5 girls) received no *Current* ICD-10 ASD diagnosis at the assessment.

Eight of the 12 children (4 boys, 4 girls) born prematurely received a *Current* diagnosis of ICD-10 Autism. Two of the prematurely born children (both boys) acquired a *Current* diagnosis of ICD-10 Atypical autism, and one child (a girl) did not fulfil criteria for a *Current* ICD-10 ASD diagnosis. In one of the prematurely born children no ICD-10 ASD diagnosis was acquired due to discontinuation of the study.

In six cases (4.8%) there had been a change in ICD-10 diagnosis when the information concerning *Ever* and *Current* symptoms acquired from the DISCO-11 interview was analysed. Four children (3 boys, 1 girl) went from an ICD-10 diagnosis of Autism to Atypical Autism, and two children (1 boy, 1 girl) changed from an ICD-10 diagnosis of Atypical Autism to No ASD diagnosis comparing the symptoms and signs over time.

The data presented in Table 2 shows that there were no significant differences in AMSE mean score, Vineland-II Parent/caregiver rating questionnaire GAF mean standard score or mean intellectual ability, across the groups of children with a *Current* ICD-10 diagnosis of Autism when they were collapsed into groups on the basis of sex and age (≤ 36 months vs ≥ 37 months of age, respectively) Table 2.

**Table 2:** AMSE mean score, Vineland II Parent/caregiver rating questionnaire GAF mean standard score, and Intellectual ability mean score for the children who acquired an ICD-10 Autism diagnosis in the study (n = 89)

Instrument	All children with ICD-10 Autism (n = 89)	Children ≤ 36 months with ICD-10 Autism (n = 14)	Girls ≤ 36 months with ICD-10 Autism (n = 5)	Boys ≤ 36 months with ICD-10 Autism (n = 9)	Children ≥ 37 months with ICD-10 Autism (n = 75)	Girls ≥ 37 months with ICD-10 Autism (n = 14)	Boys ≥ 37 months with ICD-10 Autism (n = 61)
AMSE mean (SD) Median (min;max)	9.7 (2.3) 10 (5;16)	9.6 (2.4) 9 (6;14)	9.6 (3.0) 9 (6;14)	9.7 (2.1) 9 (7;12)	9.7 (2.4) 10 (5;16)	9.3 (2.2) 9 (5;14)	9.8 (2.4) 10 (5;16)
Vineland-II GAF mean standard score (SD) Median (min;max)	66.5 (13.4) 63 (45;105) (n = 84)	66.4 (18.3) 61 (47;105) (n = 11)	67.0 (15.2) 61 (58;94)	66.0 (22.0) 59 (47;105) (n = 6)	66.5 (12.7) 65 (45;98) (n = 73)	64.1 (12.7) 62.5 (45;88)	67.1 (12.4) 65 (47; 98) (n = 59)
Intellectual level (SD) Median (min;max)	5.0 (0.9) 5 (3;6)	4.7 (0.8) 4.5 (4;6)	4.6 (0.9) 4 (4;6)	4.8 (0.8) 5 (4;6)	5.0 (0.9) 5 (3;6)	4.8 (1.0) 5 (3;6)	5.1 (0.9) 5 (3;6)

In Table 3 the results for the 124 individuals concerning AMSE, Vineland II- Parent/caregiver rating questionnaire and intellectual ability for all individuals, who concluded the study (n = 124), are presented. Individuals were collapsed into groups according to their *Current* ICD-10 ASD diagnosis or No ICD-10 ASD diagnosis. As shown in Table 3 the mean AMSE result for the ICD-10 Autism group was significantly higher compared to the ICD-10 Atypical autism (p = 0.0004) and No ICD-10 ASD Diagnosis groups (p. < 0001).

**Table 3:** AMSE mean total score, Vineland-II Parent/caregiver rating questionnaire GAF and Communication, DLS, Socialization, and Motor skills mean standard scores, and Intellectual ability mean score for all children assessed by the DISCO-11 (n = 124)

Instrument	ICD-10 Autism (A) (n = 89)	ICD-10 Atypical autism (AA) (n = 14)	ICD-10 AS* (n = 3)	No ICD-10 ASD diagnosis (n = 18)	A vs AA p value Cohens d	A vs No ASD p value Cohens d	AA vs No ASD p value Cohens d
AMSE mean score (SD) Median (min;max)	9.7 (2.3) 10 (5;16)	7.2 (2.8) 6.5 (3;11)	5.3 (1.5) 5 (4;7)	5.7 (2.0) 6 (2;8)	0.0004 0.98	<.0001 1.85	0.087 0.62
Vineland-II GAF mean standard score (SD) Median (min;max)	66.5 (13.4) 65 (45;105) (n = 84)	71.8 (14.7) 70 (48;105) (n = 13)	83.0 (20.4) 76 (67;106)	84.5 (13.6) 86 (61;120) (n = 17)	0.19 0.38	0.0001 1.33	0.018 0.90
Vineland-II Communication mean standard score (SD) Median (min;max)	64.0 (13.9) 63 (27;106) (n = 84)	71.1 (15.1) 67 (46;104) (n = 13)	81.7 (17.8) 74 (69;102)	81.6 (15.0) 82 (61;113) (n = 17)	0.093 0.49	0.0001 1.78	0.068 0.70
Vineland-II DLS mean standard score (SD) Median (min;max)	74.3 (15.1) 73 (49;121) (n = 84)	77.1 (15.4) 73 (57;113) (n = 13)	91.7 (26.6) 85 (69;121)	89.7 (14.0) 89 (67;125) (n = 17)	0.053 0.18	0.0002 1.06	0.027 0.86
Vineland-II Socialization mean standard score (SD) Median (min;max)	67.0 (13.8) 64 (46;103) (n = 84)	73.4 (12.0) 71 (58;98) (n = 13)	76.7 (15.3) 71 (65;94)	84.6 (14.4) 81 (63;117) (n = 17)	0.12 0.49	0.0001 1.25	0.032 0.84
Vineland-II Motor Skills mean standard score (SD) Median (min;max)	69.0 (15.2) 68 (42;117) (n = 84)	71.9 (16.8) 72 (42;102) (n = 13)	86.0 (16.5) 87 (69;102)	85.9 (19.0) 87 (54;114) (n = 17)	0.53 0.18	0.0001 0.98	0.044 0.78
Intellectual ability mean score (SD) Median (min;max)	5.0 (0.9) 5 (3;6)	4.9 (1.6) 6 (4;7)	6.0 (1.0) 6 (5;7)	5.5 (0.8) 5 (4;7)	0.73 0.08	0.031 0.59	0.18 0.47

Two children scoring 7, and 8 respectively on the AMSE did not require an Autism diagnosis because they did not fulfil the criteria concerning start of symptoms before the age of 3 years.

As is further visualized in Table 3 the group of children with No ICD-10 ASD diagnosis had a significantly higher mean GAF standard score on the Vineland-II Parent/caregiver rating questionnaire and domain scores compared to the ICD-10 Autism group, as well as the ICD-10 Atypical group (except for the Communication domain in the latter group).

All the groups had their strongest performance in DLS, and Communication was the domain in which all groups, with the exception of the very small AS group, had their poorest performance. The AS group had their poorest performance in Socialization.

There was a significant difference in mean intellectual ability across the groups with a *Current* ICD-10 Autism diagnosis and the No ICD-10 ASD diagnosis group.

### AMSE Item Scores

The scores for the respective items of the AMSE are presented in Table 4. All groups include both girls and boys since there was no significant difference between the subscores across the sexes. The AMSE scores were significantly higher in the ICD-10 Autism group compared to the No ASD diagnosis group for 6 of the 8 items: Eye contact, Interest in others, Pointing skills, Repetitive behaviors/stereotypy, Unusual or encompassing preoccupations and Unusual sensitivities. Of these items the Repetitive behaviors/stereotypy and Unusual or encompassing preoccupations were the items with the most pronounced significance (p < .0001).

**Table 4:** AMSE item scores for all participating children where a DISCO-11 interview was performed (n = 124)

AMSE items	ICD-10 Autism (n = 89)	ICD-10 Atypical autism (n = 14)	ICD-10 AS (n = 3)	No ICD-10 ASD (n = 18)	Autism vs Atypical autism p value	Autism vs No ASD Diagnosis p value	Atypical autism vs No ASD Diagnosis p value
Eye contact score							
0	6 (6.7%)	2 (14.3%)	0 (0.0%)	5 (27.8%)	0.17	0.0016	0.27
1	62 (69.7%)	11 (78.6%)	3 (100%)	13 (72.2%)			
2	21 (23.6%)	1 (7.1%)	0 (0.0%)	0 (0.0%)			
Interest in others score							
0	11 (12.4%)	7 (50.0%)	0 (0.0%)	6 (33.3%)	0.0020	0.0022	0.83
1	23 (25.8%)	3 (21.4%)	3 (100%)	8 (44.4%)			
2	55 (61.8%)	4 (28.6%)	0 (0.0%)	4 (22.2%)			
Pointing skills score							
0	45 (50.6%)	8 (57.1%)	3 (100%)	18 (100%)	0.33	0.0006	0.0033
1	29 (32.6%)	6 (42.8%)	0 (0.0%)	0 (0.0%)			
2	15 (16.8%)	0 (0.0%)	0 (0.0%)	0 (0.0%),			
Language score							
0	29 (32.6%)	6 (42.8%)	2 (66.7%)	8 (44.4%)	0.39	0.16	0.76
1	48 (53.9%)	7 (50.0%)	1(33.3%)	10 (55.6%)			
2	12 (13.5%)	1 (7.1%)	0 (0.0%)	0 (0.0%)			
Pragmatics of language score							
0	17 (19.1%)	1 (7.1%)	0 (0.0%)	5 (27.8%)	0.35	0.27	0.13
1	4 (4.5%)	1 (7.1%)	0 (0.0%)	2 (11.1%)			
2	68 (76.4%)	12 (85.7%)	3 (100%)	11 (61.1%)			
Repetitive behavior/stereotypy score							
0	7 (7.9%)	4 (28.6%)	2 (66.7%)	10 (55.6%)	0.056	<.0001	0.20
1	16 (18.0%)	2 (14.3%)	1 (33.3%)	1 (5.6%)			
2	66 (74.2%)	8 (57.1%)	0 (0.0%)	7 (38.9%)			
Preoccupations score							
0	12 (13.5%)	7 (50.0%)	2 (66.7%)	13 (72.2%)	0.0010	<.0001	0.28
1	56 (62.9%)	7 (50.0%)	0 (0.0%)	5 (27.8%)			
2	21 (23.6%)	0 (0.0%)	1 (33.3%)	0 (0.0%)			
Unusual sensitivities score							
0	3 (3.4%)	2 (14.3%)	3 (100%)	7 (38.9%)	0.0093	0.025	1.00
1	61 (68.5%)	12 (85.7%)	0 (0.0%)	6 (33.3%)			
2	25 (28.1%)	0 (0.0%)	0 (0.0%)	5 (27.8%)			

Three items were significantly different across the ICD-10 Autism and the ICD-10 Atypical autism groups, namely Unusual or encompassing preoccupations, Unusual sensitivities, and Interest in others. The only item that was significantly different across the ICD-10 Atypical autism and No ICD-10 ASD diagnosis groups was Pointing skills.

**Fig. 2:**
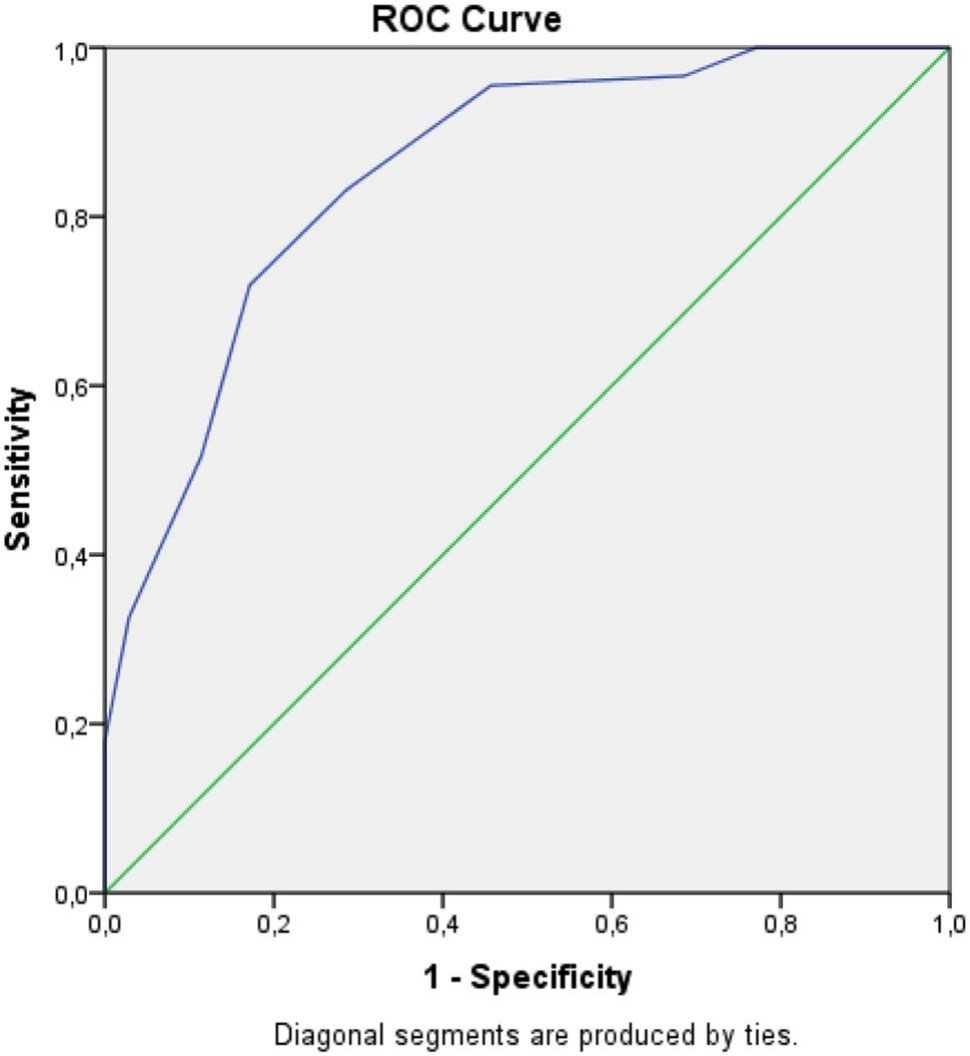
ROC curve analysis, AMSE total score × diagnosis

The scores for sensitivity, specificity, positive predictive and negative predictive values for the AMSE are presented in Table 5. The optimal cut-off level for the AMSE in this study concerning the likelihood of an ASD to be present in the child was found to be 7 p (sensitivity 0.75 and specificity 0.78, respectively), visualized by the ROC curve in Fig. 2. Sensitivity and specificity rates were considered *Fair* according to the criteria presented by Cicchetti et al. (1995).

**Table 5:** Sensitivity, specificity, positive predictive value and negative predictive value of AMSE cut-off scores for classification of ASD for the children evaluated on the DISCO 11 (n = 124)

AMSE cut-off	Sensitivity	Specificity	Positive predictive value	Negative predictive value
0.5	1.0	0.0	0.85	*
1.5	1.0	0.0	0.85	*
2.5	1.0	0.06	0.86	1.0
3.5	0.98	0.22	0.88	0.67
4.5	0.97	0.33	0.90	0.67
5.5	0.92	0.39	0.90	0.44
6.5	0.87	0.56	0.92	0.42
7.5	0.75	0.78	0.95	0.35
8.5	0.66	1.0	1.0	0.33
9.5	0.47	1.0	1.0	0.24
10.5	0.27	1.0	1.0	0.19
11.5	0.15	1.0	1.0	0.17
12.5	0.09	1.0	1.0	0.16
13.5	0.08	1.0	1.0	0.16
14.5	0.02	1.0	1.0	0.15
15.5	0.01	1.0	1.0	0.15

As visualized in Table 6 where the participating children, who acquired an ICD-10 Autism, were collapsed into groups according to their intellectual ability there were no individuals in the intellectual ability groups 1 (IQ 0–19), 2 (IQ 20–34), or 7 (IQ ≥ 120). There was a significant correlation concerning AMSE and intellectual ability, where higher intellectual ability was correlated to lower AMSE scores and vice versa (r_s_ − 0.37, p = 0.0005). The Vineland-II Parent/caregiver rating questionnaire GAF mean standard score showed a positive correlation to intellectual ability with scores increasing with higher intellectual ability (r_s_ 0.46, p < .0001). The same was true for all the domains included in the Vineland-II Parent/caregiver rating questionnaire with the strongest correlation for the Communication domain (r_s_ 0.59, p < .0001), and the weakest correlation for the Socialization domain (r_s_ 0.23, p = 0.038). In all the intellectual ability groups except for the intellectual ability group 6 the mean standard score was lower for the Communication domain than for the Socialization domain (Table 6).

**Table 6:** AMSE mean total score and Vineland-II Parent/caregiver rating questionnaire GAF mean standard score and subdomain means for children (n = 84) with an ICD-10 Autism diagnosis collapsed into groups according to intellectual ability

Instrument (IQ) (n = 89)	3 (IQ 35–49) (n = 3)	4 (IQ 50–69) (n = 26)	5 (IQ 70–89) (n = 29)	6 (IQ 90–119) (n = 31)	Spearman correlation coefficient p value
AMSE mean score (SD) Median (min;max)	11.3 (4.0) 9 (9;16)	11.0 (2.0) 11 (8;15)	9.9 (2.2) 10 (6;15)	8.4 (1.8) 9 (5;12)	r_s_ − 0.37 p = 0.0005
Vineland-II GAF mean standard score (SD) Median (min;max)	53.0 (2.0) 53 (51;55)	59.9 (9.3) 59 (45;84) (n = 23)	65.4 (13.7) 62 (47;105) (n = 28)	74.0 (12.1) 75 (49;95) (n = 30)	r_s_ 0.46 p < .0001
Vineland-II Communication mean standard score (SD) Median (min;max)	51.7 (5.5) 52 (46;57)	56.1 (10.1) 57 (39;76) (n = 23)	60.1 (12.3) 63 (27;82) (n = 28)	74.7 (11.7) 74 (52;106) (n = 30)	r_s_ 0.59 p < .0001
Vineland-II DLS mean standard score (SD) Median (min;max)	61.7 (1.2) 61 (61;63)	66.5 (9.4) 65 (49;83) (n = 23)	74.9 (17.0) 75 (55;115) (n = 28)	81.1 (14.4) 81 (49;113) (n = 30)	r_s_ 0.45 p < .0001
Vineland-II Socialization mean standard score (SD) Median (min;max)	58.7 (1.2) 58 (58;60)	65.2 (13.6) 63 (46;94) (n = 23)	66.3 (15.0) 62.5 (46;103) (n = 28)	70.0 (13.1) 69 (48;101) (n = 30)	r_s_ 0.23 p = 0.038
Vineland-II Motor skills mean standard score (SD) Median (min;max)	51.3 (2.3) 50 (50;54)	62.9 (12.6) 61 (42;91)	68.0 (14.9) 66 (50;117)	76.4 (14.7) 74 (46;114)	r_s_ 0.42 p < .0001

No children in this study were classified in intellectual ability groups 1 (IQ 0–19), 2 (IQ 20–34) or 7 (IQ ≥ 120)

### Expressive Language Ability

The results from the comparison of the groups of children diagnosed with autism using the expressive language ability levels presented on the AMSE sheet (i.e. “Nonverbal”, “Single words”, “Phrases”, “Undeveloped sentences”, and “Can speak about another time and place (presented as “Adequate speech” in Table 7)), are presented in Table 7. As can be seen in Table 7 there was a negative correlation for AMSE mean scores to expressive language ability (r_s_ − 0.42, p < .0001). The opposite was true for the GAF mean standard score where there was a positive correlation between higher GAF mean standard scores and higher expressive language ability (r_s_ 0.36, p = 0.0008). The Communication domain had the strongest correlation of the subdomains (r_s_ 0.53, p < .0001), and the Motor skills domain had the weakest correlation (r_s_ 0.24, p = 0.029). Expressive language level was positively related to age (r_s_ 0.66, p < .0001), as well as intellectual ability (r_s_ 0.53, p < .0001).

**Table 7:** Vineland II GAF mean standard score and subdomain means in relation to expressive language level in children diagnosed with ICD-10 Autism where a Vineland-II Parent/caregiver rating questionnaire was completed (n = 84)

Instrument	Nonverbal (n = 9)	Single words (n = 10)	Phrases (n = 8)	Undeve-loped sentences (n = 29)	Adequate speech* (n = 28)	Spearman correlation coefficient p value
GAF mean standard score (SD) Median (min;max)	55.0 (9.2) 51 (47;73)	66.9 (16.1) 60.5 (51;105)	65.0 (15.7) 59.5 (47;95)	65.9 (13.8) 65 (47;98)	71.2 (10.6) 74 (48;98)	r_s_ 0.36 P = 0.0008
Communication mean standard score (SD) Median (min;max)	44.4 (10.2) 44 (27;61)	59.8 (12.6) 59 (39;82)	63.9 (11.3) 62 (46;84)	63.7 (13.0) 63 (46;106)	72.0 (10.1) 74 (50;93)	r_s_ 0.53 p < 0001
DLS mean standard score (SD) Median (min;max)	63.8 (12.5) 63 (49;92)	76.0 (15.9) 74.5 (60;115)	71.5 (16.5) 67 (57;105)	74.7 (16.8) 75 (53;106)	77.6 (12.6) 77 (49;101)	r_s_ 0.28 p = 0.011
Socialization mean standard score (SD) Median (min;max)	60.8 (13.4) 60 (48;94)	68.2 (17.3) 62.5 (46;103)	66.4 (20.2) 61.5 (46;101)	66.9 (13.8) 63 (46;101)	69.0 (10.3) 69 (51;88)	r_s_ 0.24 p = 0.027
Motor skills mean standard score (SD) Median (min;max)	62.6 (12.8) 61 (42;80)	70.8 (20.8) 65 (46;117)	65.2 (14.3) 59 (50;91)	66.0 (12.6) 65 (46;91)	74.5 (15.5) 72 (50;114)	r_s_ 0.24 p = 0.029
AMSE mean standard score (SD) Median (min;max)	11.7 (3.4) 12 (6;16)	10.2 (2.3) 9.5 (8;14)	11.0 (2.4) 10 (9;15)	9.8 (1.4) 10 (7;14)	8.3(1.8) 8.5 (5;12)	r_s_ − 0.42 p < .0001
Intellectual ability mean score (SD) Median (min;max)	4.2 (0.6) 4 (3;5)	4.3 (0.8) 4 (3;6)	5.1 (0.8) 5 (4;6)	4.9 (0.8) 5 (4;6)	5.6 (0.7) 6 (3;6)	r_s_ 0.53 p < .0001
Mean age mean in months (SD) Median (min;max)	38.2 (9.6) 37 (21;56)	39.0 (10.6) 37.5 (23;55)	45.8 (9.2) 48 (32;56)	59.1 (12.3) 59 (28;79)	64.6 (10.6) 63.5(43;94)	r_s_ 0.66 p < .0001

*Adequate speech equals the “Can speak about another time and place” language level on the AMSE sheet

The “Nonverbal” and “Single words” group of children had a DLS > Motor skills > Socialization > Communication profile. In contrast, the language groups “Phrases” and “Undeveloped sentences” had a DLS > Socialization > Motor skills > Communication profile, and the “Can speak about another time and place” (Adequate speech) group had a DLS > Motor skills > Communication > Socialization profile.

When the children diagnosed with ICD-10 Autism were collapsed into groups according to chronological age (≤ 36, 37–48, 49–60, 61–72, ≥ 73 months, respectively), as visualized in Table 8, the only significant correlation was that AMSE mean scores decreased with age (r_s_ − 0.23, p = 0.038)

**Table 8:** Vineland II GAF mean standard score and subdomain means in relation to age in children with ICD-10 Autism where Vineland-II Parent/caregiver rating questionnaire was completed (n = 84)

Instrument	≤ 36 (n = 11)	37–48 (n = 14)	49–60 (n = 28)	61–72 (n = 17)	≥ 73 (n = 14)	Spearman correlation coefficient p value
GAF mean standard score (SD) Median (min–max)	66.4 (18.3) 61 (47;105)	62.7 (16.0) 56 (45;95)	66.5 (11.0) 68 (47;88)	72.5 (13.0) 75 (48;98)	63.3 (10.6) 62.5 (47;81)	r_s_ 0.16 p = 0.14
Communication standard score (SD) Median (min;max)	54.4* (23.5) 46 (27;106)	62.1*(14.9) 59(44;93)	65.1*(10.9) 63(46;84)	70.7 (9.8) 72 (52;91)	62.9*(9.4) 63(48;78)	r_s_ 0.26 p = 0.018
DLS standard score (SD) Median (min;max)	78.3 (19.3) 74 (55;115)	67.7 (15.6) 63 (49;105)	72.9 (10.0) 74 (57;93)	83.1 (17.8) 81 (53;121)	70.2 (12.2) 68 (49;97)	r_s_ 0.13 p = 0.23
Socialization standard score (SD) Median (min;max)	66.4 (14.6) 61 (50;103)	65.4 (15.2) 61 (48;101)	67.4 (14.0) 66 (46;94)	70.6(15.0) 71 (48;101)	64.1 (10.0) 65 (48;81)	r_s_ 0.12 p = 0.26
Motor skills standard score (SD) Median (min;max)	74.8 (19.4) 72 (46;117)	65.4 (18.9) 57 (42;114)	68.6 (14.9) 66 (46;99)	68.7*(19.9) 69 (50;84)	69.2 (14.7) 67 (50;91)	r_s_ 0.06 p = 0.56
AMSE mean score (SD) Median (min;max)	9.2 (2.5) 8 (6;14)	11.9 (2.3) 12 (9;14)	9.7 (2.1) 10 (5;14)	8.9 (1.2) 9 (7;11)	8.6 (2.2) 9 (5;11)	r_s_ − 0.23 p = 0.038
Intellectual ability mean score (SD) Median (min;max)	4.8 (0.9) 5 (4;6)	4.6 (1.0) 4.5 (3;6)	5.0 (0.8) 5 (4;6)	5.4 (0.9) 6 (3;6)	5.2 (0.9) 5(3;6)	r_s_ 0.29 p = 0.0080

*Indicating the subdomain with lowest standard score for each age group

## Discussion

Of the 124 children concluding the study there were 81 boys, and 25 girls, who had a *Current* ICD-10 ASD diagnosis giving a 3.2:1 male:female ratio, which is a sex ratio in the lower interval compared to the data presented by Loomes et al. (2017), who had found a male:female sex ratio of 3.1–4.3:1 in their meta-analysis of sex differences in ASD. In the present study the individuals having an intellectual ability within the normal intellectual distribution (i.e. ≥ IQ 70) had a male:female ratio of 4.4:1 compared to a 1.9:1 male:female ratio for the children with an intellectual ability below IQ 70. The lower sex ratio for children with intellectual disability found in this study was in line with earlier findings (e.g. Fombonne et al. 2009; Kim et al. 2011).

The results from the present study supports earlier findings concerning the validity of the AMSE as an instrument in the process of diagnosing ASD. In this study the AMSE mean score was significantly higher for the group of children who acquired a *Current* ICD-10 diagnosis of Autism, compared to children who did not fulfill criteria for a *Current* ICD-10 ASD diagnosis at the assessment.

In this study the optimal cut-off level for a probable ASD was found to be a score of 7 p on the AMSE, with sensitivity, and specificity scores of 75%, and 78%, respectively acquiring a *Fair* reliability according to the criteria by Cicchetti et al. (1995). Grodberg et al. (2012) found an AMSE score of 5p to be the optimal cut-off level concerning sensitivity as well as specificity for the AMSE in their study. In this study none of the children scoring below 5p acquired an ICD-10 ASD diagnosis, however children with an AMSE score as high as 8p did not acquire an ICD-10 ASD diagnosis. In two cases were children scored 7, and 8p respectively, on the AMSE, the criteria for symptom start before the age of 3 years was not fulfilled. If the recently published ICD-11 criteria had been used in this study these two children would have acquired an Autism diagnosis since the ICD-11 does not require the start of autism symptoms before the age of 3 years (World Health Organisation 2018). However, even if the AMSE pinpoints crucial symptoms and signs present in an ASD there are of course a number of other symptoms and signs required for an ICD-10 ASD diagnosis, which might at least partly explanation why participants scoring above the cut-off score of 7 point on the AMSE still did not acquire an ICD-10 ASD diagnosis in this study. In addition, the group of children who did not receive an ICD-10 ASD diagnosis in the study had a significantly higher GAF mean standard score than the group of children who received an ICD-10 ASD diagnosis, indicating that a good functioning in daily life activities might conceal difficulties required for sufficient ICD-10 ASD diagnosis criteria to be met. Furthermore, the mean intellectual ability was somewhat, albeit not, significantly higher in the No ASD diagnosis group than the group of children who fulfilled criteria for an ICD-10 ASD diagnosis. Szatmari et al. (2015) have reported that higher intellectual functioning was related to a later acquired ASD diagnosis in their study, which might also be true for some of the individuals in the No ICD-10 ASD diagnosis group in the present study. In the present study we found AMSE to be negatively correlated to higher intellectual ability, as well as to expressive language level and age, which further supports the findings by Szatmari et al. (2015).

The AMSE item scores were significantly higher in 6 of the 8 items for the children with a ICD-10 Autism diagnosis compared to the children with No ICD-10 ASD diagnosis, with Pointing skills, Repetitive behaviors/stereotypy, and Unusual or encompassing preoccupations being the items with the most significant difference across the groups.

There was no significant difference in AMSE mean score across the groups of children above and below 36 months of age, however the total maximum score for the younger group was 14 points compared to 16 points in the older groups since item 5 (odd intonation and/or difficulties in taking turns) was considered difficult to score reliably in the younger group. Hence, these results points in the direction that more autistic symptoms and signs have to be present in younger children for them to be referred for neuropsychiatric assessment.

In the present study the AMSE scores were not significantly different across the groups of boys and girls which stands in contrast to the findings of Øien et al. (2018a, b), who reported of significant differences in the scores for items 4 “Language” (girls scored significantly higher) and 8 “Unusual sensitivities“(girls scored significantly lower) from their study. In contrast to the study by Øien et al. (2018b), Tillmann et al. (2018) did not find specific sex-related differences in their multi-centre study, which is supported by the findings in this study.

The Vineland-II GAF mean standard scores had a positive correlation to intellectual ability. All the intellectual ability groups, with the exception of the group of children with the highest intelligence score, had a Communication mean standard score which was lower than their Socialization mean standard score, corresponding to earlier research in the field of autism (e.g. Fernell et al. 2010).

In this study all expressive language groups had their best performance in DLS, which is not supported by earlier findings. The “Nonverbal” and “Single words” groups of children assessed in the present study had a DLS > Motor skills > Socialization > Communication profile, which stands in contrast to the reports from Sparrow et al. 2005, who in their original assessment of the Vineland-II Parent/caregiver rating questionnaire, found a Motor skills > DLS > Socialization > Communication profile for non-verbal children and adolescents, findings confirmed by other authors (e.g. Paul et al. 2014; Ray-Subramanian et al. 2011). The expressive language groups “Phrases” and “Undeveloped sentences” had a DLS > Socialization > Motor skills > Communication profile in the present study, and the expressive language group “Can speak about another time and place” (Adequate speech) had a DLS > Motor skills > Communication > Socialization profile in comparison to the reported profiles from Sparrow et al. (2005), who reported profiles for verbal children to be Motor skills > Communication > DLS > Socialization. However, other studies on toddlers and preschool children (verbal and nonverbal) with ASD reported a Motor skills > DLS > Communication > Socialization profile (e.g. Yang et al. 2016; Andersson et al. 2013). There is no clear explanation for the different Vineland-II Parent/caregiver rating questionnaire profiles acquired from the present study, compared to earlier research. However, the mean intellectual ability in the investigated group was in the lower normal range (IQ 70–89), which is a relatively high intellectual level for a group of children with ASD, and this might explain the relatively better functioning in DLS for the children in in the present study.

This study supported the findings by Yang et al. (2016) that the children with the lowest intellectual ability had relatively higher Vineland-II GAF mean standard scores, and individuals with the highest intellectual ability had lower Vineland-II mean standard scores than would be expected considering their respective intellectual ability.

Six children (4.8%) had enough reduction in autistic symptoms on the DISCO-11 interview for a change in ICD-10 diagnosis comparing the *Ever* to *Current* situations, which is in line with earlier research by Lord et al. (2006) who reported that most changes in symptom severity in their study occurred during the pre-school years. Szatmari et al. (2015) reported from a multicentre study in Canada that approximately 11% of their cohort had less symptoms of autism as measured by ADOS at age 6 compared to baseline at approximately 40 months of age. In addition, Moss et al. (2008) found in their study that children who have better language skills and higher adaptive behavior skills at pre-school years were more likely to show improvement in symptom severity.

Finally, since the participants in this study was acquired from children referred for neuropsychiatric assessment based on the concern of caregiver(s) and/or medical professionals, the base-rate of ASD symptoms in the present sample is therefore likely to be higher than in clinical samples based on positive level-one screening, and this selection bias is important to note as it might impact the observed psychometric properties of the AMSE.

## Conclusion

The results from the present study speaks in favour of the AMSE as a valid instrument in the identification of ASD in pre-school children referred for neuropsychiatric assessment in a clinical setting. The AMSE scores had a *Fair* correlation to ICD-10 ASD diagnoses acquired from an interview with parent(s)/caregiver(s) using the DISCO-11. The AMSE has advantages compared to other assessment/screening instruments since it is easy to administer, score and evaluate. In addition, it is less time-consuming compared to other instruments, albeit at the same time more informative since it includes information acquired by the professional at the clinical assessment, as well as information from the parent(s)/caregiver(s). Earlier studies have found a good correlation of AMSE towards ADOS-2, ADI-R, and DSM-5 ASD diagnosis, which further enhance the usefulness of this instrument as a tool in the assessment of children suspected of having an ASD. Hence, it has every opportunity to become a basic assessment tool in the clinical setting as well as in research in the future.

## Limitation

Since this is a reasonably small study including 126 pre-school children further studies are required to further assess the usefulness of the AMSE in research as well as in the clinical setting in the screening and diagnostic process of pre-school children suspected of having an ASD.

## References

[CR1] American Psychiatric Association (2013). Diagnostic and Statistical manual of mental disorders.

[CR2] Anderssson G, Gillberg G, Miniscalco C (2013). Pre-school children with suspected autism spectrum disorders: Do girls and boys have the same profiles?. Research in Developmental Disabilities.

[CR3] Baron-Cohen S, Allen J, Gillberg C (1992). Can Autism be detected at 18 months? The needle, the haystack and the CHAT. British Journal of Psychiatry.

[CR4] Bayley N (2006). Bayley scales of infant and toddler development.

[CR5] Cicchetti DV, Volkmar F, Klin A, Showalter D (1995). Diagnosing Autism using ICD-10 criteria. A comparison of neural networks and standard multivariate procedures. Child Neuropsychology.

[CR6] Ehlers S, Gillberg C, Wing L (1999). A screening questionnaire for Asperger Syndrome and other high-functioning autism spectrum disorders in school age children. Journal of Autism and Developmental Disorders.

[CR7] Fernell E, Hedvall A, Norrelgen F, Eriksson M, Höglund-Carlsson L, Barnevik-Olsson M (2010). Developmental profiles in preschool children with Autism Spectrum Disorders referred for intervention. Research in Developmental Disabilities.

[CR8] Fombonne E (2009). Epidemiology of pervasive developmental disorders. Pediatric Research.

[CR9] Grodberg D, Siper P, Jamison J, Buxbaum JD, Kolevzon A (2016). A simplified diagnostic observational assessment of autism spectrum disorders in early childhood. Autism Research.

[CR10] Grodberg D, Weinger PM, Halpern D, Parides M, Kolevzon A, Buxbaum JD (2014). The autism mental status exam: sensitivity and specificity using DSM-5 criteria for Autism Spectrum Disorder in verbally fluent adults. Journal of Autism and Developmental Disorders.

[CR11] Grodberg D, Weinger PM, Kolevzon A, Soorya L, Buxbaum JD (2012). Brief report: The Autism mental status examination: Development of a brief autism-focused exam. Journal of Autism and Developmental Disorders.

[CR12] Kim YS, Leventhal BL, Koh Y-L, Fombonne E, Laska E, Lim E-C (2011). Prevalence of Autism Spectrum Disorders in a total population sample. American Journal of Psychiatry.

[CR13] Krug DA, Arick J, Almond P (1980). Behavior checklist for identifying severely handicapped individuals with high levels of autistic behaviours. Journal of Child Psychology and Psychiatry.

[CR14] Landis JR, Koch GG (1977). The measurement of observer agreement for categorical data. Biometrics.

[CR15] Le Couteur A, Rutter M, Lord C, Rios P, Robertson S, Holdgrafer M, Mc Lennan J (1989). Autism diagnostic interview: a standardized investigator-based instrument. Journal of Autism and Developmental Disorders.

[CR16] Loomes R, Hull L, Polmear Locke Mandy W (2017). What is the male-to-female ratio in Autism Spectrum Disorder? A systematic review and meta-analysis. Journal of the American Academy of Child and Adolescent Psychiatry.

[CR17] Lord C, Luyster RJ, Gotham K, Guthrie W (2012). Autism diagnostic observation schedule, second edition (ADOS-2) manual (part II): Toddler module.

[CR18] Lord C, Risi S, DiLavore PS, Shulman C, Thurm A, Pickles A (2006). Autism from 2 to 9 years of age. Archives of General Psychiatry.

[CR19] Lord C, Rutter M, DiLavore PC, Risi S (2001). Autism diagnostic observational schedule.

[CR20] Lord C, Rutter M, DiLavore PC, Risi S, Gotham K, Bishop S (2012). Autism diagnostic observation schedule, second edition (ADOS-2) manual (Part I): modules I–IV.

[CR21] Lord C, Rutter M, LeCouteur A (1994). Autism diagnostic interview-revised: a revised version of a diagnostic interview for caregivers of individuals with possible pervasive developmental disorders. Journal of Autism and Developmental Disorders.

[CR22] Maljars M, Noens I, Scholte E, Berckelaer-Onnes I (2012). Evaluation of the criterion and convergent validity of the diagnostic interview for social and communication disorders in young and low functioning children. Autism.

[CR23] Moss J, Magiati J, Charman T, Howlin P (2008). Stability of the Autism Diagnostic Interview-Revised from Pre-school to Elementary School Age in Children with Autism. Journal of Autism and Developmental Disorders.

[CR24] Nygren G, Hagberg B, Billstedt E, Skoglund Å, Gillberg C, Johansson M (2009). The swedish version of the diagnostic interview for social and communication disorders. psychometric properties. Journal of Autism and Developmental Disorders.

[CR25] Øien RA, Schjølberg S, Volkmar FR, Shic F, Cicchetti DV, Nordahl-Hansen A (2018). Clinical features of children with autism who passed 18-month screening. Pediatrics.

[CR26] Øien R, Vambheim S, Hart L, Nordahl-Hansen A, Erickson C, Wink l (2018). Sex differences in children referred for assessment: An exploratory analysis of the Autism Mental Status Exam (AMSE). Journal of Autism and Developmental Disorders.

[CR27] Paul R, Loomis R, Chawarska K (2014). Adaptive behavior in toddlers under two with autism spectrum disorders. Journal of Autism and Developmental Disorders.

[CR28] Posserud M, Lundervold A, Gillberg C (2009). Validation of the autism spectrum screening questionnaire in a total population sample. Journal of Autism and Developmental Disorders.

[CR29] Ray-Subramanian CE, Huai N, Ellis Weismer S (2011). Brief report: Adaptive behavior and cognitive skills for toddlers on the autism spectrum. Journal of Autism and Developmental Disorders.

[CR30] Robins DL, Casagrande K, Barton M, Chen C-MA, Dumont-Mathieu T, Fein D (2014). Validation of the modified check-list for autism in toddlers, revised with follow-up (M-CHAT R/F). Pediatrics.

[CR31] Robins DL, Fein D, Barton ML, Green JA (2001). The Modified Checklist for Autism in Toddlers: an initial study investigating the early detection of autism and pervasive developmental disorders. Journal of Autism and Developmental Disorders.

[CR32] Roid G, Sampers R (2000). Merrill-palmer scale revised.

[CR33] Rutter M, Bailey A, Berument SK, Le Couteur A, Lord C, Pickles A (2003). Social communication questionnaire (SCQ).

[CR34] Sparrow S, Cicchetti D, Balla D (2005). Vineland adaptive behavior scales-2nd Edition Manual.

[CR35] Szatmari P, Georgiades S, Duku E, Bennett TA, Bryson S, Fombonne E (2015). Developmental trajectories of symptom severity and adaptive functioning in an inception cohort of preschool children with autism spectrum disorder. JAMA Psychiatry.

[CR36] Tillmann J, Ashwood K, Absoud M, Bölte S, Bonnet-Brilhaut F, Buitelaar JK (2018). Evaluating Sex and Age Differences in ADI-R and ADOS scores in a large European Multi-Site sample of individuals with Autism Spectrum Disorder. Journal of Autism and Developmental Disorders.

[CR37] Wechsler D (2002). Wechsler preschool and primary scale of intelligence.

[CR38] Wechsler D (2003). Wechsler intelligence scale for children.

[CR39] Wechsler D (2012). Wechsler preschool and primary scale of intelligence.

[CR40] Wechsler D, Naglieri JA (2006). Wechsler nonverbal scale of ability.

[CR41] Wing, L. (2006). *Diagnostic Interview for Social and Communication Disorders (11th Ed.)* Bromley, UK: Centre for Social and Communication Disorders.

[CR42] Wing L, Gould J (1978). Systematic recording of behaviors and skills of retarded and psychotic children. Journal of Autism and Childhood Schizophrenia.

[CR43] Wing L, Leekam SR, Libby SJ, Gould J, Larcombe M (2002). The diagnostic interview for social and communication disorders: Background, inter-rater reliability and clinical use. Journal of Child Psychology and Psychiatry.

[CR44] World Health Organisation (1992). Manual of the International statistical classification of diseases, and related health problems-Tenth Revision (ICD-10).

[CR45] World Health Organisation (2018). Manual of the International statistical classification of diseases, and related health problems-Eleventh Revision (ICD-11).

[CR46] Yang S, Paynter JJ, Gilmore L (2016). Vineland adaptive behavior scales: II profile of young children with autism spectrum disorders. Journal of Autism and Developmental Disorders.

